# Diverse functional roles of miR-128-3p in human diseases: Focus on its roles in human cancer

**DOI:** 10.1016/j.gendis.2025.101736

**Published:** 2025-06-26

**Authors:** Lingzi Zheng, Sheng Yan, Jinling Zhang, Weisen Ning, Xiaoliu Liu, Xiaomei Wang, Ling Hu

**Affiliations:** a Tianyou Hospital, Affiliated to Wuhan University of Science and Technology, Wuhan, Hubei 430064, China; b College of Medicine, Wuhan University of Science and Technology, Wuhan, Hubei 430065, China; c Wuhan Fourth Hospital, Wuhan Orthopedic Hospital, Wuhan 430033, China; d NHC Key Laboratory of Nuclear Technology Medical Transformation, Manyang Central Hospital, Manyang, Sichuan 621000, China

**Keywords:** Cancer, Diagnostic markers, MicroRNA, MiR-128-3p, Pathway

## Abstract

The microRNA known as miR-128-3p demonstrates widespread tissue-specific expression with its genetic locus situated on human chromosome 2 (2q21.3), and is a multifunctional regulator with tissue-specific expression patterns that critically influences cellular homeostasis and disease pathogenesis. By engaging with the 3′ untranslated region (3′ UTR) of target mRNA molecules, miR-128-3p exquisitely modulates gene expression levels, thereby orchestrating cellular proliferation, immune responses, metabolic equilibrium, and tumorigenesis. Studies on miR-128-3p in different cancers have revealed diverse expression patterns and functional roles. Intriguingly, due to its ability to target multiple genes and signaling pathways, as well as being regulated by various genes themselves, miR-128-3p exhibits both tumor-suppressive and oncogenic effects under neoplastic conditions. This review summarizes the expression patterns and complex regulatory mechanisms of miR-128-3p across various cancers. A profound understanding of the significance and regulation of miR-128-3p in cancer will drive the development of innovative therapeutic strategies using this molecule for combating human cancer and immune-related disorders.

## Introduction

Non-coding RNAs (ncRNAs) constitute a diverse class of RNA molecules that play critical roles in regulating gene expression without encoding proteins. These ncRNAs operate through various mechanisms, including chromatin modification, transcriptional interference, and post-transcriptional control.^1^ This broad category encompasses long non-coding RNAs (lncRNAs), circular RNAs (circRNAs), and microRNAs (miRNAs), each exhibiting distinct regulatory functions. Among these, miRNAs have been recognized as pivotal regulators due to their precise role in post-transcriptional gene silencing.^2^

miRNAs have garnered significant attention for their ability to finely tune gene expression at the post-transcriptional level.^3^ The biogenesis of miRNAs commences in the nucleus, where RNA polymerase II transcribes primary miRNAs (pri-miRNAs).^4^ These pri-miRNAs are subsequently processed by the Drosha complex into precursor miRNAs (pre-miRNAs) of approximately 70 nucleotides, which are then exported to the cytoplasm via Exportin-5.^5^ In the cytoplasm, Dicer further cleaves pre-miRNAs to produce mature miRNAs of about 22 nucleotides.^6^ These mature miRNAs are incorporated into the RNA-induced silencing complex (RISC), where they recognize target mRNAs through complementary seed sequences, leading to translational repression or mRNA degradation. Consequently, miRNAs modulate essential biological processes such as cellular differentiation, proliferation, apoptosis, and tumorigenesis.^7^, ^8^, ^9^

In contrast to miRNAs, lncRNAs and circRNAs typically utilize more complex structural mechanisms to regulate gene expression. For example, lncRNAs can scaffold chromatin-modifying complexes or sequester transcription factors, while circRNAs frequently function as competitive endogenous RNAs (ceRNAs) by sponging miRNAs or interacting with RNA-binding proteins.^10^^,^^11^ However, miRNAs remain uniquely advantageous for translational research due to their direct regulatory mechanisms, stability under physiological conditions (attributed to their small size and protection within protein complexes), and capacity for systemic delivery. The dysregulation of miRNAs is increasingly acknowledged as a hallmark of cancer pathogenesis. These molecules can act as tumor suppressors or oncogenes (oncomiRs), directly influencing malignant phenotypes such as uncontrolled proliferation, metastatic dissemination, and therapeutic resistance.^12^

Their exceptional stability in biofluids, compared to the rapid degradation of mRNAs by ribonucleases, positions miRNAs as promising non-invasive biomarkers for cancer diagnosis and prognosis. Furthermore, miRNA-based therapeutic strategies, including mimic restoration for tumor-suppressive miRNAs and antagomir-mediated oncogenic miRNA inhibition, are advancing toward clinical validation.

Of particular interest is miR-128-3p, a member of the miR-128 family, which has recently gained prominence as a multifunctional regulator. Emerging studies have begun unraveling its context-dependent roles in tumorigenesis, immune modulation, and therapeutic response, underscoring its potential as both a diagnostic biomarker and a therapeutic targe.^13^ Notably, among the myriad miRNAs, miR-128-3p, a pivotal member of the miR-128 family, has recently emerged as a key regulator with its biological functions and underlying mechanisms progressively being elucidated.

**Figure 1:**
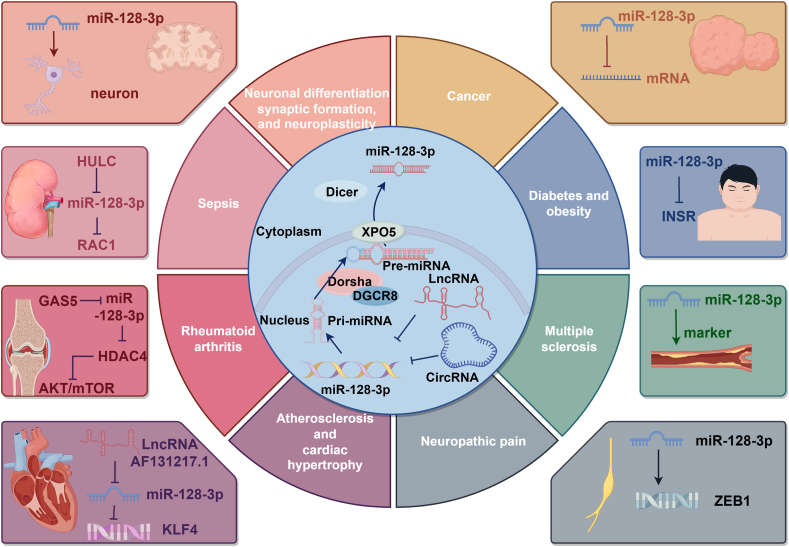
miR-128-3p participates in a variety of physiological and pathological processes in the human body.

The gene encoding miR-128-3p is located on human chromosome 2 (2q21.3), where its precursor miRNA (pre-miRNA) is cleaved by the Dicer enzyme to generate mature miR-128-3p within cells. Mature miR-128-3p is a highly conserved 22-nucleotide sequence (5′-UCACAGUGAACCGGUCUCUUU-3′). This microRNA is broadly expressed across various tissues and cell types, including the brain, heart, liver, and lungs. Notably, miR-128-3p is highly expressed in the brain, playing crucial roles in neuronal differentiation, synapse formation, and neuroplasticity.^14^ Additionally, the involvement of miR-128-3p in the cardiovascular system has garnered significant attention, as it contributes to the occurrence and progression of diseases such as atherosclerosis and cardiac hypertrophy by regulating the functions of vascular endothelial cells and smooth muscle cells.^15^ Furthermore, miR-128-3p is implicated in the regulation of the insulin signaling pathway, influencing glucose metabolism and lipogenesis, which is particularly relevant to metabolic diseases such as diabetes and obesity.^16^ It also alleviates respiratory inflammation by enhancing mitochondrial function in asthma.^17^ In addition to these roles, miR-128-3p is associated with various diseases, including multiple sclerosis,^18^ neuropathic pain,^19^ cellular sepsis,^20^ and rheumatoid arthritis.^21^ Additionally, miR-128-3p is involved in several physiological processes, including inflammation,^22^ tissue damage,^23^ and cell proliferation and differentiation^24^ (Fig. 1). In summary, miR-128-3p not only participates in a diverse range of physiological and pathological activities but also functions as a gene regulator, exerting either oncogenic or tumor-suppressive roles during tumor initiation and progression by binding to the untranslated regions of mRNAs and targeting specific genes.

## Functional roles of miR-128-3p in human cancers

The pattern of miR-128-3p expression in human cancer has been extensively studied. The expression of miR-128-3p is down-regulated in non-small cell lung cancer (NSCLC) tissues,^25^, ^26^, ^27^ as well as the serum exosomal miR-128-3p levels.^28^ Interestingly, some studies have reported an up-regulation of miR-128-3p in NSCLC tissue and lung squamous cell carcinoma.^29^^,^^30^ Furthermore, miR-128-3p exhibits a strong correlation with tumor node metastasis (TNM) stage, smoking status, and tumor size in lung cancer tissues. Notably, miR-128-3p, either alone or in combination with miR-33a-5p, demonstrates superior diagnostic performance compared to traditional tumor markers such as CYFR21-1, NSE, and CA72-4.^26^ Moreover, exosomal miR-128-3p shows promise in the screening of NSCLC, with combined detection alongside miR-33a-5p yielding increased sensitivity.^28^ High levels of miR-128-3p expression are associated with poor prognosis and are identified as an independent prognostic factor for patients with NSCLC.^31^ However, some studies have indicated that the overall survival of NSCLC patients with low expression of miR-128-3p is significantly reduced.^27^ This discrepancy highlights the necessity for further investigation into the potential of miR-128-3p as a promising biomarker for the early detection of lung cancer.

In hepatocellular carcinoma (HCC), down-regulation of miR-128-3p has been observed.^32^^,^^33^ A decreased level of miR-128-3p serves as an independent predictor of shorter disease-free survival in liver cancer patients; those with low miR-128-3p expression exhibit decreased disease-free survival rates following liver cancer resection.^34^ Additionally, miR-128-3p demonstrates inhibitory effects on the expression of CYP2C9 in HCC,^35^ and CYP2C9 has been identified as a potential prognostic marker for HCC.^36^ These findings suggest that miR-128-3p may hold promise as a valuable prognostic indicator for HCC.

In the cancer genome atlas (TCGA) database, miR-128-3p is significantly overexpressed in breast cancer tissue compared to normal breast tissue, with elevated levels of miR-128-3p being associated with improved survival expectancy in breast cancer patients.^37^ Similarly, analysis of the GSE59829 dataset revealed a significant up-regulation of miR-128-3p in metastatic breast cancer patients relative to non-metastatic cases, where higher miR-128-3p expression was correlated with reduced distant metastasis-free survival (DMFS) in breast cancer patients.^38^ However, evidence suggests that both breast cancer tissues and cell lines exhibit diminished expression of miR-128-3p,^39^ which has been corroborated in patients with advanced breast cancer.^40^ Furthermore, in the context of breast cancer, miR-128-3p expression is correlated with reduced disease-free survival.^41^ These findings imply that the regulation of miR-128-3p expression in breast cancer is influenced by multiple factors and does not follow a uniform pattern of up-regulation or down-regulation, potentially varying with tumor stage. Therefore, further investigations are warranted to determine the potential of miR-128-3p as a diagnostic or prognostic biomarker in breast cancer.

**Figure 2:**
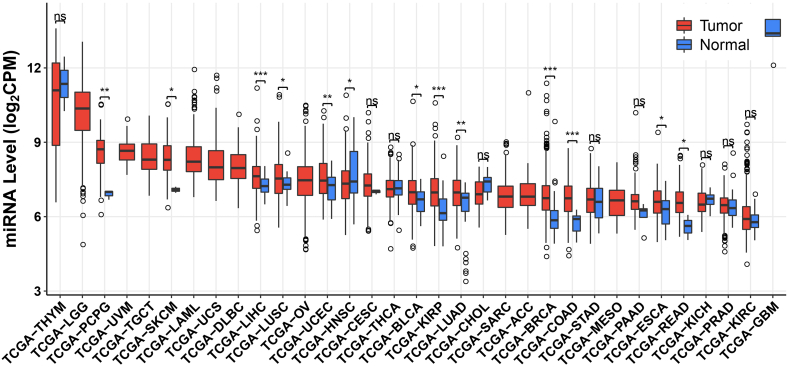
Pan-cancer analysis of miR-128-3p expression.^46^

Additionally, miR-128-3p is down-regulated in human glioblastoma multiforme (GBM) tissues.^42^, ^43^, ^44^ In colorectal cancer, exosomes derived from HCT-116 cells induced by epithelial–mesenchymal transition (EMT) exhibit high expression levels of miR-128-3p. Clinically, increased expression of miR-128-3p is significantly associated with perineural invasion, lymphovascular invasion, tumor stage and CA 19-9 content in patients with colorectal cancer.^45^ Figure 2 presents a comprehensive pan-cancer analysis of miR-128-3p expression, revealing its differential regulation across various malignancies and further substantiating its role as a promising diagnostic biomarker.^46^ MiR-128-3p expression is aberrant in tissues and cells in different systemic cancers, suggesting that it might be used as a tumor diagnostic marker.

## The molecular mechanism underlying miR-128-3p functions in cancers

miR-128-3p is implicated in a complex regulatory network, where it is modulated by various factors in cancer and regulates multiple genes and signaling pathways. Here, we systematically summarize all the regulatory situations associated with miR-128-3p and its impact on different types of cancer (Table 1, Table 2).

**Table 1:** Upstream regulators influencing the expression of miR-128-3p.

Transcription factor/lncRNA	Effect on transcription	Cancer
IL18R1^30^	Repression	Lung cancer
LINC00346^55^	Repression	Glioma
FOXD3-AS1^56^^,^^68^	Repression	Glioma, CC
SNHG22,^57^ ASLNC07322^58^	Repression	CRC
LINC00467^59^		
Smad4^58^	Activation	CRC
TGFβ1^62^	Repression	Breast Cancer
PVT1^63^^,^^44^	Repression	Breast Cancer, Glioma
DLEU2^65^	Repression	CC
OIP5-AS1^67^	Repression	OC
MIR4435-2HG^69^^,^^70^	Repression	OC, CC
lncRNA MIAT^25^^,^^78^	Repression	OS, Lung cancer
SNHG16^79^, ^80^, ^81^	Repression	RB, GIST, and NB

Abbreviations: CC, cervical carcinoma; CRC, colorectal cancer; OC, ovarian cancer; MM, multiple myeloma; OS, osteosarcoma; RB, retinoblastoma; GIST, gastrointestinal stromal tumor; NB, neuroblastoma; PCa, prostatic cancer; PC, pancreatic cancer.

**Table 2:** Identified targets of miR-128-3p in human cancers.

Implicated cancer	Target	Effect
Lung cancer	Drosha and Dicer^29^	Promotion of cancer metastasis
	PELI3^25^	Promotion of metastasis and proliferation
	CCND1^31^	Overactivation of Wnt/β-catenin and TGF-β pathway
	GATA2^47^	Enhance RhoA pathway
	ADAM28^27^	Inactivation of JAK2/STAT3 pathway
	ABCG2, CTR2^31^	Resistance to cisplatin, gemcitabine, and paclitaxel
	SKA3^48^	Tumor suppression
	TGF-β, VEGFC^49^	Promotion of sensitivity to Coptisine
	SPTAN1^50^	Weakening DNA inter-strand cross-link repair and promoting chromosomal aberrations
HCC	c-Met^32^	Enhancing the sensitivity of lenvatinib-resistant HCC cells to Lenvatinib
	DJ-1^33^	Enhancing the sensitivity to Sorafenib
	p85α^34^	Activation of PIK3R1 and PI3K/AKT pathway
	CYP2C9^35^	To be researched
	CDC6^51^	Inducing cell G0-G1 phrase arrest
Glioma	GREM1^44^	Activation of BMP pathway
	c-Met,^42^ WEE1^56^	Enhancing the sensitivity to temozolomide
	NPTX1^43^	Activation of PI3K/AKT pathway
	PDK1^54^	Disrupting mitochondrial function
	SZRD1^55^	Tumor suppression
CRC	E2F3,^57^ VEGFC^58^^,^^59^	Tumor suppression
	NPTX1^60^	Silence of PI3K/AKT and MEK/ERK pathway
Breast cancer	NEK2^61^	Tumor suppression and inhibition of cancer stemness
	LIMK1^37^	Tumor suppression
	MET,^63^ FOXQ1^63^	Inhibition of cancer migration and invasion
CC	CCNG1,^67^ LIMK1,^68^ MSI2,^70^ ZEB1^72^	Tumor suppression
OC	CDK14^69^	Tumor suppression
MM	NTRK3^73^	Tumor suppression
MM	PLAGL2^76^	Tumor suppression
OS	VEGFC^78^	Tumor suppression
GIST	CASC3^80^	Tumor suppression
ESCC	ZEB1^82^	Inhibition of cancer migration and invasion

Abbreviations: HCC, hepatocellular carcinoma; ESCC, Esophageal squamous cell carcinoma; GC, gastric carcinoma.

## Lung cancer

In non-small cell lung cancer, miR-128-3p restores the invasion, migration and colony formation ability of cancer cells inhibited by MIAT deletion by targeting PELI3.^25^ Meanwhile, miR-128-3p promoted the migration of lung cancer cells by down-regulating the expression of the miRNA-processing enzymes Drosha and Dicer.^29^ In lung squamous cell carcinoma, the down-regulation of the T-cell cytotoxic marker IL18R1 promotes the up-regulation of miR-128-3p, which subsequently promotes the proliferation and migration of cancer cells.^30^ Moreover, miR-128-3p can up-regulate the expression of cyclin D1 (CCND1), resulting in excessive activation of the Wnt/β-catenin and TGF-β pathways that promote non-small cell lung cancer progression. Moreover, miR-128-3p induces the expression of the drug transporters ATP binding cassette transporter G2 (ABCG2) and copper transporter 2 (Ctr2), thereby increasing the resistance to cisplatin, gemcitabine and paclitaxel in NSCLC cells while enabling their survival under these chemotherapeutic treatments with larger and more numerous colonies. The overexpression of miR-128-3p significantly enhances the self-renewal ability in NSCLC and increases the proportion of cancer stem cells.^31^ Furthermore, miR-128-3p can down-regulate GATA binding protein 2 (GATA2) to enhance the RhoA pathway, thus promoting lung cancer cell proliferation, migration, and invasion.^47^

However, several studies have indicated that miR-128-3p exerts inhibitory effects on the progression of lung cancer cells. Specifically, miR-128-3p suppresses the proliferation, migration, and invasion of cancer cells by targeting spindle and kinetochore associated complex subunit 3 (SKA3) and promoting apoptosis in NSCLC.^48^ MiR-128-3p directly targets A disintegrin and metalloproteinase 28 (ADAM28) and inhibits its expression, leading to inactivation of the JAK2/STAT3 signaling pathway, thereby inhibiting autophagy and promoting NSCLC cell apoptosis.^27^ Moreover, through downregulating TGF-β and VEGFC expression levels, miR-128-3p enhances NSCLC cell sensitivity to Coptisine.^49^ Additionally, mitomycin C (MMC) is an effective DNA cross-linking agent that is used as a chemotherapeutic drug because it causes DNA inter-strand cross-link in cancer cells. MiR-128-3p can attenuate DNA inter-strand cross-link repair by targeting spectrin alpha non-erythrocytic 1 (SPTAN1), leading to cell cycle arrest in the G2/M phase and promoting chromosomal aberrations in MMC-treated lung cancer cells.^50^

To sum up, miR-128-3p exhibits a dual regulatory effect on lung cancer by concurrently promoting cell growth and inhibiting cell proliferation. Hence, further investigations are warranted to elucidate the biphasic mechanism of miR-128-3p in lung cancer.

## Hepatocellular carcinoma

MiR-128-3p regulates the cell cycle of hepatocellular carcinoma (HCC) cells by targeting the cellular-mesenchymal epithelial transition factor (c-Met), thereby attenuating the down-regulation of ERK and cyclin D1 induced by lenvatinib in Huh7 cells. Additionally, it regulates the Akt-mediated apoptosis pathway, thereby enhancing the sensitivity of lenvatinib-resistant HCC cells to lenvatinib treatment.^32^ The overexpression of miR-128-3p can promote sorafenib-induced cell apoptosis by suppressing DJ-1 expression and increasing the sensitivity of HCC cells to sorafenib through the PTEN/PI3K/Akt pathway.^33^ Moreover, miR-128-3p induces G0–G1 phase arrest in HCC cells, inhibiting their growth via down-regulation of p85α and CDC6, with p85α regulating the activation of the PI3K/Akt pathway.^34^^,^^51^ Furthermore, miR-128-3p mediates the down-regulation of CYP2C9, a crucial drug-metabolizing enzyme (DME), which is involved in the metabolism of various carcinogens and drugs in HCC.^35^ However, further investigations are warranted to elucidate how miR-128-3p affects CYP2C9 inhibition in HCC. Chronic hepatitis B virus X protein (HBx) amplifies TRPM7-mediated calcium influx by sponging miR-128-3p, subsequently activating JNK signaling via the TRPM7/Ca^2+^/JNK pathway. Briefly, miR-128-3p participates in the HBx-induced carcinogenesis and doxorubicin resistance through the TRPM7/Ca^2+^/JNK signaling pathway.^52^ The long non-coding RNA HLA-F antisense RNA 1 (HLA-F-AS1) promotes the proliferation and migration of HCC cells by sequestering miR-128-3p.^53^ In conclusion, while functioning as a tumor-suppressive gene in HCC, paradoxically it attenuates drug resistance in liver cancer cells to various drugs.

## Glioma

The overexpression of miR-128-3p that is sponged by the lncRNA PVT1 results in the down-regulation of Gremlin 1 (GREM1), through the BMP signaling pathway, inducing G0–G1 phase arrest, which subsequently inhibits glioma cell proliferation, migration, and invasion while promoting apoptosis.^44^ MiR-128-3p targets c-Met to inhibit glioma cell proliferation and suppresses EMT-related proteins and pathways to impede the migration and invasion of glioblastoma cells. Additionally, miR-128-3p enhances the chemosensitivity of glioblastoma to temozolomide (TMZ).^42^ It has been demonstrated that miR-128-3p negatively regulates neuronal pentraxin 1 (NPTX1). Transfection of glioma cells with a miR-128-3p mimic resulted in elevated levels of phosphorylated insulin receptor substrate 1 (IRS1), phosphoinositide 3 kinase (PI3K), and p-AKT, accompanied by reduced cell proliferation rates.^43^ Pyruvate dehydrogenase kinase 1 (PDK1) is significantly up-regulated in glioma tissues. Silencing PDK1 expression leads to decreased lactate and ATP production, accumulation of ROS, mitochondrial damage, slowed cell growth, and increased apoptosis. Suppression of PDK1 induced by miR-128-3p increases pyruvate entry into the TCA cycle within mitochondria, producing large amounts of ROS, thereby facilitating apoptosis in glioblastoma cells through disruption of mitochondrial function.^54^ The up-regulation of long intergenic non-protein-coding RNA 346 (LINC00346) is associated with poor prognosis in gliomas; its down-regulation inhibits GBM cell proliferation while acting as a ceRNA for miR-128-3p. Suz RNA binding domain containing 1(SZRD1), a downstream target gene regulated by miR-128-3p, is up-regulated by LINC00346 through sponging miR-128-3p, thereby promoting tumor cell proliferation.^55^ FOXD3-ASl, a lncRNA recently discovered to be overexpressed in gliomas and correlated with poor prognosis among patients, also participates in GBM resistance to TMZ treatment. By acting as a sponge for miR-128-3p, FOXD2-ASl increases WEE1 expression and enhances tolerance to TMZ.^56^ The role of miR-128-3p in gliomas extends beyond its direct or indirect anti-cancer effects, as it also enhances sensitivity to temozolomide, thereby showcasing its multifaceted therapeutic potential.

## Colorectal cancer

**Figure 3:**
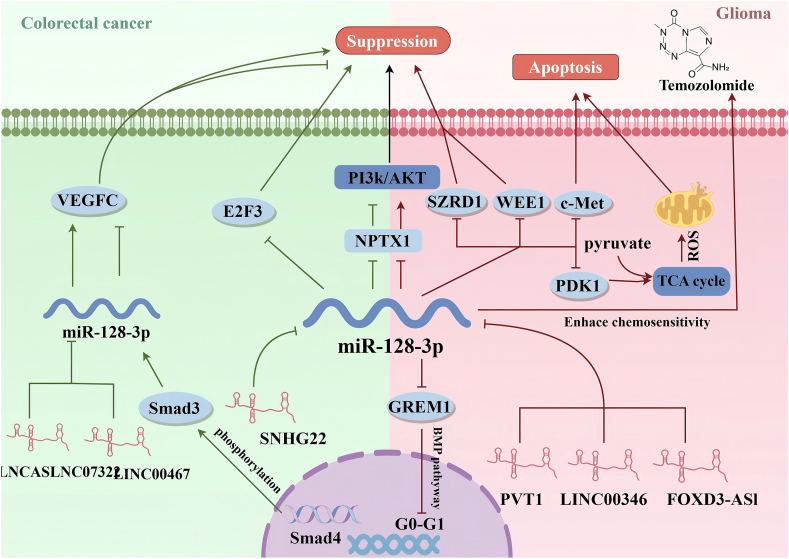
In various cancers, similar regulatory mechanisms modulate miR-128-3p, exerting either tumor-suppressive or oncogenic effects. (VEGFC, vascular endothelial growth factor C; E2F3, E2F transcription factor 3; NPTX1, neuronal pentraxin 1; SZRD1, Suz RNA binding domain containing 1; c-Met, cellular-mesenchymal epithelial transition factor; PDK1, pyruvate dehydrogenase kinase 1; Smad3, SMAD family member 3; GREM1, Gremlin 1; ASLNC07322, long noncoding RNA ASLNC07322; LINC00467, long nonprotein coding RNA 467; SNHG22, lncRNA small nucleolar RNA host gene 22; Smad4, SMAD Family Member 4; PVT1, lncRNA PVT1; LINC00346, long noncoding RNA 346; FOXD3-AS1, non-coding RNA forkhead box D3 antisense RNA 1.)

High expression levels of the lncRNA small nucleolar RNA host gene 22 (SNHG22) are significantly associated with advanced clinicopathological factors and a poor survival rate in colorectal cancer patients. The knockdown of SNHG22 markedly inhibited the proliferation, anti-apoptotic properties, migration, and invasion of CRC cells *in vitro*, and suppressed tumor growth *in vivo*. SNHG22 functions as a sponge for miR-128-3p, diminishing its inhibitory effect on the expression and activity of E2F transcription factor 3 (E2F3), thereby promoting the occurrence and development of CRC cells.^57^ Deletion and mutation of Smad4 favor colon cancer progression while negatively correlating with vascular endothelial growth factor C (VEGFC) regulation. The overexpression of Smad4 enhances the phosphorylation of Smad3, facilitating its translocation into the nucleus, where it binds to the promoter of miR-128-3p and promotes its transcription. Subsequently, miR-128-3p negatively regulates VEGFC expression by binding to its 3’ untranslated region (UTR). This pathway plays a dominant role in non-metastatic colorectal cancer. Interestingly, the lncRNA ASLNC07322 is specifically up-regulated in metastatic colon cancer and functions as a sponge for miR-128-3p, leading to an increase in VEGFC. Both Smad4 and ASLNC07322 simultaneously influence miR-128-3p by down-regulating or up-regulating VEGFC expression, which promotes or inhibits tumors respectively.^58^ Additionally, the expression level of long intergenic non-protein coding RNA 467 (LINC00467) was significantly elevated in both colorectal cancer tissues and cell lines. The down-regulation of LINC00467 expression in CRC cells can effectively suppress cell proliferation, metastasis and angiogenesis. LINC00467 competitively binds to miR-128-3p and up-regulates VEGFC, thereby mediating the growth, migration, and invasion of CRC cells.^59^ Furthermore, miR-128-3p can inhibit the progression of colorectal cancer by targeting NPTX1 and silencing both the PI3K/AKT and MEK/ERK pathways.^60^ It remains to be determined whether metastatic colorectal cancer influences the expression of miR-128-3p, which appears to play a tumor suppressor role in colorectal cancer. In both colorectal cancer and glioma, miR-128-3p affects the development of cancer by targeting NPTX1 (Fig. 3).

## Breast cancer

The overexpression of miR-128-3p inhibits the stem-like characteristics of breast cancer stem cells by inhibiting the Wnt signaling pathway by down-regulating NEK2.^61^ Under 24 h of hypoxic treatment, the expression of miR-128-3p significantly increased in MCF-10A cells but decreased in invasive MDA-MB-231 cells. The low levels of miR-128-3p provide an advantage for MDA-MB231 cells against hypoxic stress. Compared to luminal MCF-7 cells, the basal cell line MDA-MB-231 displayed greater sensitivity to treatment with either miR-128-3p or empagliflozin (EMPA).^40^ LIM domain kinase 1 (LIMK1) is a direct target gene of miR-128-3p, while cofilin 1 is a downstream target of LIMK1. The overexpression of miR-128-3p leads to decreased expression levels of both LIMK1 and cofilin 1 (CFL1), thereby inhibiting breast cancer cell proliferation. Furthermore miR-128-3p overexpression inhibits the migration and invasion of breast cancer cells by reducing the expression levels of MMP2 and MMP9. MiR-128-3p can also arrest breast cancer cells in the G0/G1 phase by modulating the expression of CDK4/CDK6/Cyclin D1 and CDK2/Cyclin E1 ^37^. TGFβ signaling plays a critical role in breast cancer progression and is specifically elevated during the metastatic transformation of aggressive breast cancer. TGFβ1 triggers hepatocyte growth factor (HGF)-induced and MET-dependent migration *in vitro*. MET is a direct target of miR-128-3p, which is negatively regulated by TGFβ1. The overexpression of miR-128-3p reduces MET expression and abolishes HGF-induced migration of invasive breast cancer cells.^62^ PVT1 is significantly up-regulated in both plasma samples and cell lines derived from breast cancer patients, promoting the proliferation and metastasis of breast cancer cells both *in vivo* and *in vitro*. Mechanistically, PVT1 acts as a competing endogenous RNA for miR-128-3p, resulting in increased FOXQ1 expression and promotion of epithelial–mesenchymal transition.^63^ Notably, the up-regulation of miR-128-3p by empagliflozin contributes to increased ferroptosis and reduced metastasis, specifically in stromal-separated cells.^64^ Overall, the expression of miR-128-3p plays a tumor suppressor role in breast cancer, which is also influenced by cell type.

## Gynecologic cancer

The expression of Deleted In Lymphocytic Leukemia 2 **(**DLEU2) is up-regulated in cervical carcinoma (CC). Down-regulation of DLEU2 can arrest the cell cycle of cervical cancer cells at the G2/M phase by up-regulating miR-128-3p, thereby inhibiting cervical cancer cell proliferation *in vitro*, inducing apoptosis, and suppressing tumor growth *in vivo*.^65^ Circ_0007142 acts as a sponge for miR-128-3p, leading to the downregulation of S100 calcium binding protein A14 (S100A14), which inhibits the malignant behavior of CC cells.^66^ The expression of o-phthalaldehyde interacting protein 5 antisense transcript 1 (OIP5-AS1) is elevated in ovarian cancer tissues and cells. OIP5-AS1 up-regulates CCNG1 by functioning as a sponge for miR-128-3p, promoting the occurrence and progression of ovarian cancer.^67^ The expression of long non-coding RNA forkhead box D3 antisense RNA 1 (FOXD3-AS1) is increased in cervical cancer tissues and cell lines compared to that in adjacent healthy tissues and normal cervical epithelial cells. FOXD3-AS1 targets and negatively regulates miR-128-3p, thereby indirectly up-regulating LIMK1 expression and promoting CC progression.^68^ Both the miR4435-2 host genes (miR4435-2HG and CDK14) are overexpressed in ovarian cancer (OC) tissues and cells. MiR4435-2HG competitively binds to miR-128-3p to up-regulate CDK14, and the knockdown of miR4435-2HG can inhibit the development of OC cells through the miR-128-3p/CDK14 axis.^69^ Additionally, miR4435-2HG is significantly up-regulated in both cervical cancer tissues and cells, and its knockout can inhibit the proliferation, migration and invasion of CC cells.^70^ The expression level of miR-128-3p is lower in endometrial cancer tissues than in adjacent tissues and is correlated with adverse clinicopathological parameters in patients, suggesting its potential as a prognostic marker and therapeutic target for this disease.^71^ Circ-ABCB10 displays high expression levels in cervical cancer tissues and cells. Loss of circ-ABCB10 significantly impairs the proliferation and invasion capabilities of CC cells by inhibiting EMT processes and inducing apoptosis. Specifically, circ-ABCB10 acts as a competitive endogenous RNA by binding to miR-128-3p. Consequently, the depletion of circ-ABCB10 results in the up-regulation of miR-128-3p, which directly targets zinc finger E-box binding homeobox 1 (ZEB1), thereby suppressing its expression and hindering the progression of cervical cancer.^72^ These findings indicate that miR-128-3p serves as a critical tumor suppressor in gynecological malignancies.

## Other cancers

miR-128-3p is expressed at low levels in the malignant melanoma cell line NTRK3, an oncogene that enhances the malignant behavior of malignant melanoma cells and is up-regulated in these cells. MiR-128-3p acts as a tumor suppressor by inhibiting the proliferation, migration, and invasion of malignant melanoma cells and inducing apoptosis via the down-regulation of NTRK3.^73^ Gypenoside LI, a monomer derived from Gynostemma Pentaphyllum, can up-regulate miR-128-3p to inhibit the proliferation of melanoma cells through the Wnt/β-catenin signaling pathway.^74^

In nasopharyngeal carcinoma, the overexpression of miR-128-3p targets the inhibition of VEGFC expression, significantly reducing the proliferation of 6–10B and C666-1 cells. This leads to DNA damage and apoptosis induction while enhancing radiosensitivity through the P50/P65/IκB signaling pathway.^75^ HCP5 is significantly elevated in multiple myeloma (MM). HCP5 regulates PLAGL2 expression by sponging miR-128-3p, thereby participating in the occurrence and development of MM through the Wnt/β-catenin/cyclin D1 signaling pathway.^76^ This regulatory pathway is also present in nasopharyngeal carcinoma.^77^ The lncRNA MIAT is significantly up-regulated in osteosarcoma (OS) tissues and cell lines. Through its interaction with miR-128-3p, MIAT enhances VEGFC expression, thereby promoting OS progression.^78^

In neuroblastoma (NB) tissues and cells, SNHG16 and HOXA7 expression is significantly increased while miR-128-3p expression is significantly decreased. The knockout of SNHG16 can inhibit the proliferation, migration, and invasion and induce the apoptosis of NB cells by down-regulating HOXA7 via the up-regulation of miR-128-3p.^79^ SNHG16 is overexpressed in gastrointestinal stromal tumor (GIST) cells and is regulated by CTCF. MiR-128-3p is negatively regulated by SNHG16, and CASC3 is a direct target mRNA of miR-128-3p. CTCF exerts a functional effect on the malignant behavior of GIST cells through the SNHG16/miR-128-3p/CASC3 axis.^80^ Additionally, SNHG16 is also frequently up-regulated in retinoblastoma tissues and cell lines, where it exerts its carcinogenic activity by sponging miR-128-3p.^81^ ZEB1 plays a crucial role in EMT. Downregulation of miR-128-3p expression is observed in esophageal squamous cell carcinoma (ESCC) tissues and cells, leading to the inhibition of EMT and metastasis through ZEB1 down-regulation. Furthermore, down-regulation of miR-128-3p expression is associated with poor prognosis in ESCC patients, serving as an independent prognostic factor.^82^

## Conclusion and outlook

**Figure 4:**
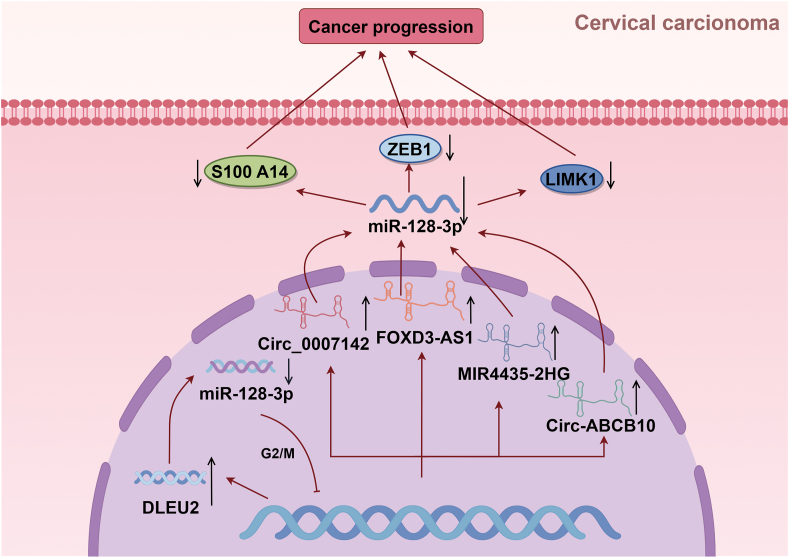
In the same type of cancer, distinct regulatory mechanisms modulate miR-128-3p, thereby exerting either tumor-suppressive or oncogenic effects. (S100A14, S100 calcium binding protein A14; ZEB1, zinc finger E-box binding homeobox 1; LIMK1, LIM domain kinase 1; DLEU2, deleted in lymphocytic leukemia 2; Circ_0007142, circular RNA 0007142; FOXD3-AS1, long noncoding RNA forkhead box D3 antisense RNA 1; MIR4435-2HG, miR4435-2 host gene; Circ-ABCB10, circular RNA ABCB10.)

Emerging evidence highlights the context-dependent duality of miR-128-3p in oncogenesis, with its expression patterns and functional roles exhibiting marked heterogeneity across cancer types. By regulating target genes and signaling pathways, miR-128-3p plays a pivotal role in tumorigenesis and development, making it a potential therapeutic target for various cancers. To enhance its inhibitory effect on colorectal cancer growth while avoiding exposure to degrading enzymes in the blood, nanocomposites loaded with miR-128-3p have been developed.^60^ As an important microRNA, studies on the expression and functional properties of miR-128-3p across various cancers provide new insights into the occurrence and development of cancer. Although its role varies across different types of cancer, there is an overall trend suggesting that through targeting specific signaling pathways and genes, miR-128-3p significantly influences the biological behavior of cancer cells (Fig. 4). Its detailed mechanism has been particularly elucidated in cases of lung and liver cancer, demonstrating its potential as a treatment option for these malignancies. However, challenges remain in miR-128-3p research. For instance, differences observed in its expression levels among studies indicate a need for more comprehensive research to clarify its specific roles across diverse cancers. Furthermore, although promising as a diagnostic or therapeutic target alone, combining miR-128-3p with other molecular markers yields more substantial effects than its use independently. Future studies should focus on revealing the specific molecular mechanism of miR-128-3p′s actions across diverse cancers while evaluating its clinical value as both a diagnostic tool and therapeutic target. Through multi-center and large-scale clinical trials, the biomarker potential of miR-128-3p can be further validated, facilitating the development of precision treatment strategies centered on miR-128-3p. In conclusion, investigating the role of miR-128-3p in cancer not only deepens our understanding of cancer biology but also paves new avenues for future cancer diagnosis and therapy.

## CRediT authorship contribution statement

**Lingzi Zheng:** Writing – original draft, Writing – review & editing, Formal analysis, Resources. **Sheng Yan:** Writing – review & editing, Formal analysis, Visualization, Writing – original draft, Data curation. **Jinling Zhang:** Investigation, Writing – original draft, Formal analysis, Writing – review & editing, Funding acquisition. **Weisen Ning:** Writing – original draft. **Xiaoliu Liu:** Writing – review & editing, Conceptualization, Visualization. **Xiaomei Wang:** Investigation, Project administration, Conceptualization, Writing – review & editing, Funding acquisition. **Ling Hu:** Project administration, Conceptualization, Writing – review & editing, Funding acquisition, Investigation.

## Funding

This study was supported by the Natural Science Foundation of Hubei, China (No. 2023AFB1111), the Open Project Fund of Hubei Key Laboratory of Embryonic Stem Cell Research (China) (No. 2022ESOF009), the Wuhan Health Commission Gene Funding Project (China) (No. WX21C25), and the NHC Key Laboratory of Nuclear Technology Medical Transformation (Mianyang Central Hospital) (China) (No. 2024HYX001).

## Conflict of interests

The authors declare no conflict of interests.
